# Radiolabeling of Nanoparticles and Polymers for PET Imaging

**DOI:** 10.3390/ph7040392

**Published:** 2014-04-02

**Authors:** Katharina Stockhofe, Johannes M. Postema, Hanno Schieferstein, Tobias L. Ross

**Affiliations:** Institute of Nuclear Chemistry, Johannes Gutenberg-University Mainz, Fritz-Strassmann-Weg 2, 55128 Mainz, Germany; E-Mails: stockhofe@uni-mainz.de (K.S.); postema@uni-mainz.de (J.M.P.); schiefe@uni-mainz.de (H.S.)

**Keywords:** positron emitter, radiolabeling, nanoparticles, polymers, drug delivery, PET, imaging, nanomedicine

## Abstract

Nanomedicine has become an emerging field in imaging and therapy of malignancies. Nanodimensional drug delivery systems have already been used in the clinic, as carriers for sensitive chemotherapeutics or highly toxic substances. In addition, those nanodimensional structures are further able to carry and deliver radionuclides. In the development process, non-invasive imaging by means of positron emission tomography (PET) represents an ideal tool for investigations of pharmacological profiles and to find the optimal nanodimensional architecture of the aimed-at drug delivery system. Furthermore, in a personalized therapy approach, molecular imaging modalities are essential for patient screening/selection and monitoring. Hence, labeling methods for potential drug delivery systems are an indispensable need to provide the radiolabeled analog. In this review, we describe and discuss various approaches and methods for the labeling of potential drug delivery systems using positron emitters.

## 1. Introduction

The field of nanomedicine has attracted more and more interest over the last decades, as nanoparticles (NPs) and polymeric structures have been related to biological and pathophysiological questions. Originally, NPs or polymers were designed and used for various purposes, like magnetic resonance imaging (MRI), computed tomography (CT) and optical imaging or simply drug delivery [1,2]. Therefore, these materials had to meet different requirements with respect to their applications. On the other hand, NPs and polymers provide an almost ideal platform to combine different modalities such as the combination of drug delivery with functional imaging techniques such as positron emission tomography (PET) or single photon computed tomography (SPECT). In this line, molecular imaging modalities are essential for patient screening/selection and monitoring in personalized therapy approaches. Furthermore, non-invasive molecular imaging techniques are excellent tools to investigate pharmacological profiles and to identify the optimal nanodimensional architecture of the aimed-at drug delivery system. Multimodal hybrid technologies such as PET/CT or PET/MRI were developed giving the chance to examine the pharmacological profiles of NPs or polymers [3]. Additionally, polymer-drug conjugates, which have already been applied for chemotherapy approaches, were radiolabeled to investigate their biodistribution via PET imaging [4]. PET, with its possibility to detect and quantify picomolar amounts of a radiotracer, has emerged as one of the most powerful imaging techniques, which underlines its outstanding role for functional imaging of physiological and/or pathophysiological processes.

**Figure 1:**
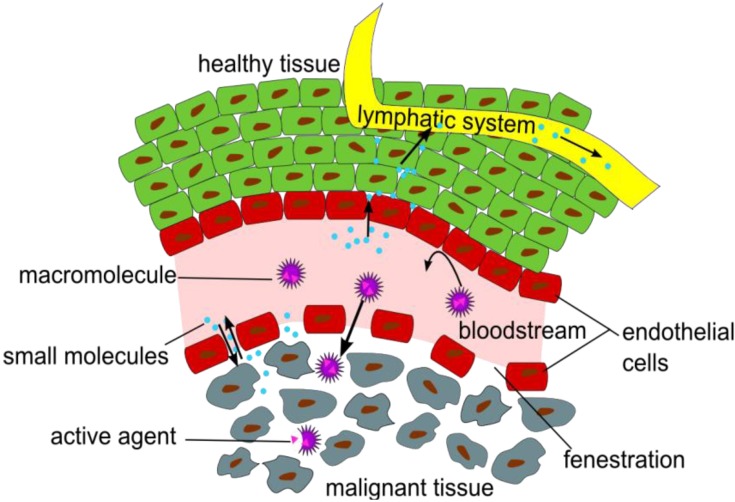
Illustration of the Enhanced Permeation and Retention (EPR) effect of macromolecular structures as drug delivery systems in malignant tissue.

Thus, novel strategies were explored for the radiolabeling of NPs (inorganic and organic) or polymers to investigate their pharmacodynamics and pharmacokinetics *in vivo* in dependence on their architecture, size and structure. To investigate the *in vivo* characteristics of NPs and polymers, it has to be considered how they are interacting with tissues and cells, and especially which time frame allows a suitable visualization of certain effects and functions, like the enhanced permeability and retention (EPR) effect (Figure 1), which is a passive targeting phenomenon and the mostly used mechanism for the uptake of NP or polymers at oncological target sites in pre-clinical and clinical studies [5,6].

The EPR effect describes the accumulation of NPs in tumor tissues, due to fenestrations in the blood vessel’s endothelial layer and a significantly reduced lymphatic drainage in the tumor tissue [7].

Since this review focuses on radiolabeling strategies for polymers and NPs for PET imaging, the radionuclides discussed are positron emitters. Radiolabeling strategies for NPs and polymers using other radionuclides for SPECT or endoradiotherapy are reviewed elsewhere [8,9].

**Figure 2:**
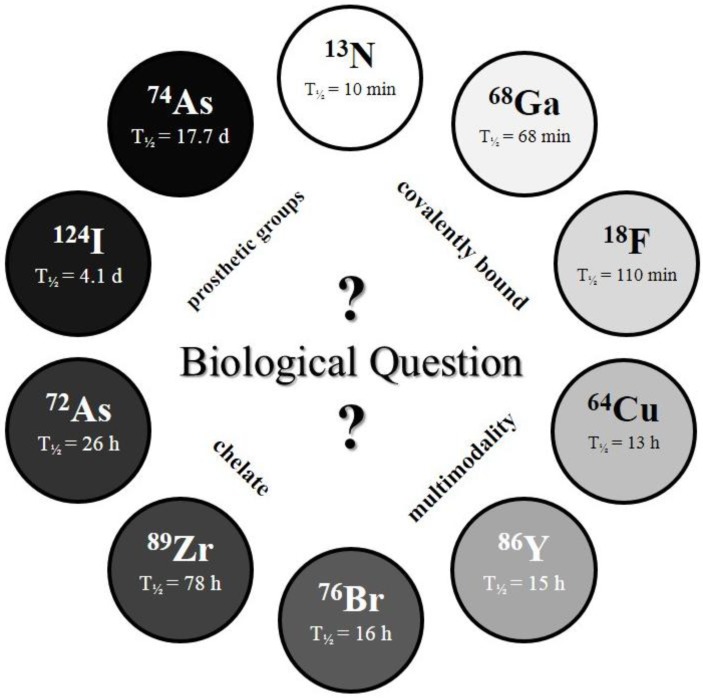
The *Clock-Of-Nuclides* showing the positron emitters used for radiolabeling of NPs or polymers, so far. Clockwise starting at ^13^N (at noon) with the shortest physical half-life and ending at ^74^As with the longest physical half-life.

The physical half-life (T_½_) of the radionuclide plays a crucial role for measurements in the desired time frame, and it has to be considered which radionuclide or half-life, respectively, is suitable for the investigated question and pharmacokinetic profile (*c.f.* Figure 2).

For measurements within a short (initial) time frame after intravenous administration, short-lived radionuclides have been applied, e.g., fluorine-18 (T_½_ = 109.7 min), gallium-68 (T_½_ = 67.7 min) or interestingly even nitrogen-13 (T_½_ = 9.97 min) [4,10,11]. Exemplarily, fluorine-18, which asks for covalent attachment to the NPs or polymers, is the most widely used PET nuclide and therefore, ^18^F-labeling strategies are of high interest. Due to its ideal imaging characteristics and good availability, ^18^F is a very attractive radionuclide for radiolabeling of NPs and polymers. Even if the accumulation, using the EPR effect, is a relatively slow process, first indications about a potential renal clearance or fast metabolism of the NPs or polymers can be obtained by using short-lived radionuclides such as ^18^F. On the other hand, the multifarious types of NPs and polymers require different coupling strategies, which guarantee fast, stable and high yielding of the respective radiosynthesis/radioconjugation. Numerous possibilities of how a radionuclide can be attached to these systems (either directly or via prosthetic group labeling) are available and essential. In case of ^18^F, direct radiolabeling is often impossible or provides only low radiochemical yields (RCYs). Consequently, an alternative (indirect) labeling strategy has to be considered, frequently resulting in novel coupling reactions using (novel) ^18^F-labeled prosthetic groups.

In contrast to the short-lived radionuclides, radiolabeling with longer-lived radionuclides allows a prolonged time frame for scanning. Examples are copper-64 (T_½_ = 12.7 h), bromine-76 (T_½_ = 16.2 h), iodine-124 (T_½_ = 4.1 d) and arsenic-74 (T_½_ = 17.8 d) [12,13,14]. Moreover, it has to be considered if the NP or polymer allows radiolabeling *via* covalent linkage (e.g., using radioiodine) or *via* a bifunctional chelator (BFC) (e.g., using radiometals). Thus, labeling with a radiometal requires a chelator, which forms stable complexes with the radiometal. The most widely used chelators are 1,4,7,10-tetra-azacyclododecane-1,4,7,10-tetraacetic acid (DOTA) and 1,4,7-triazacyclononane-1,4,7-triacetic acid (NOTA) as examples of macrocyclic chelating agents. Further prominent examples are acyclic chelators like diethylenetriaminepentaacetic acid (DTPA) and deferoxamine (DFO). Additionally, some of the chelating systems enable a *theranostic* approach by substituting the diagnostic radionuclide with a therapeutic one, whereas the chelator and the nanodimensional structure remain. Furthermore, it is possible to couple, e.g., NPs, which are MR-active to a chelating system enabling *in vivo* tracking by multimodal imaging techniques (e.g., PET/MRI).

Most of the radiolabeled NPs and polymers were subsequently tested *in vivo* to explore which systems can ultimately serve as for which imaging modality in personalized/individualized therapy approaches. So far, the aim of predominantly preclinical studies is to develop several tools for potential therapy approaches of malignancies and to overcome the current limitations in availability of suitable and individualized (radiolabeled) drug delivery systems.

This review article will summarize information about radiolabeling procedures of NPs or polymers intended for PET imaging and their potential use as drug delivery systems. Additionally, first *in vitro*/*in vivo* data are briefly discussed. Furthermore, this article should reveal how the biological question determines the radionuclide selection. In detail, every section has a short introduction followed by a summary table of already used approaches, which includes the type of nanostructure, material, size range of nanostructure, obtained specific activity, reaction time and RCY. Subsequently, the summarized data are discussed and unique characteristics of the radiolabeling and *in vivo* behavior are highlighted.

## 2. ^18^F-Labeling Approaches for Nanoparticles and Polymers

^18^F is the most commonly used positron emitter and its optimal positron energy (E_β+,max_) (635 keV) and high β^+^ intensity (97%) are almost ideal for PET imaging. Its half-life of 109.8 min allows for even extensive radiosyntheses and enables shipment of ^18^F-radiopharmaceuticals or ^18^F itself. In spite of all these benefits, in the clinics, ^18^F is mostly used in the form of 2-[^18^F]fluoro-2-deoxy-d-glucose ([^18^F]FDG). Recently, ^18^F has been used as a suitable PET nuclide to track NPs, quantum dots (QDs) or polymers, *in vivo* [4,15,16,17,18,19,20,21,22,23,24]. Section 2 will give a short overview of the research carried out using ^18^F-labeling in combination with polymers and NPs and their most important characteristics.

As shown in Table 1, several different systems have been radiolabeled with ^18^F over the last years. The short half-life of ^18^F has, however, undoubtedly limited the number of labeling studies performed. Nevertheless, the early biodistribution data of the first four hours post-injection (p.i.) are crucial with respect to the initial excretion routes and seem to give a good idea of the general pharmacokinetic profile and the potential as a drug delivery system.

**Table 1:** An overview of ^18^F-labeled nanoparticles, polymers and their important characteristics. (n.d. = no data, RCY = radiochemical yield, h.r. = hydrodynamic radii, HPMA = *N*-(2-hydroxypropyl)methacrylamide, DSPE-PEG2000-NH_2_ = 1,2-distearoyl-*sn*-glycero-3-phosphoethanolamine-*N*-[amino(polyethylene glycol)-2000], Cd = cadmium, Se = selenium, Zn = zinc, S = sulfur, Na = sodium, Y = yttrium, F = fluorine, Yb = ytterbium, Er = erbium, Tm = terbium, Gd = gadolinium, Ce = cerium, O = oxygen, Al = aluminium, Si = silicium).

Nanostructure/system	Material	Size [nm]	Specific activity	Reaction time [min]	RCY [%]	Ref.
phospholipid coated core/shell quantum dot	CdSe/CdZnS DSPE-PEG2000-NH_2_	≥20	37–75 MBq/nmol	145	n.d	[15]
nanoparticles	NaYF_4_ (co-doped with Yb, Er, Tm, Gd)	10–20	n.d.	10 (only labeling)	92	[16]
nanoparticle/peptide	gold/CLPFFD (peptide)	23 (h.r.)	27 atoms ^18^F per NP ^*^	60 (only labeling)	0.3–0.8	[17]
amino functionalized nanoparticle	CeO_2_ (ceria)	5	n.d	n.d	17.7 ± 0.3	[18]
hydrophobin functionalized porous silicon	p-type porous silica	215 ± 54	73.4 ± 13.9 MBq/g	10 (only labeling)	40.2 ± 0.5	[19]
nanoparticles	Al_2_O_3_ (alumina)	n.d	2.3 ± 0.2 MBq/mg	6 (only irradiation)	n.d	[20]
nanoparticles	gold	n.d	n.d	n.d	n.d	[21]
nanoparticles	mesoporous SiO_2_ (silica)	100–150	n.d	n.d	70	[22]
polymers	HPMA-based block copolymers	n.d	1.5–2.5 MBq/μmol	n.d	≥50	[4]
polymers	HPMA-based block copolymers	n.d	n.d	n.d	10–37	[23]
polymers	HPMA-based block copolymers	38–113 (h.r.)	n.d	n.d	5–18	[24]

* The authors calculated this value from the radioactivity-to-mass-ratio.

### 2.1. ^18^F-Labeled Quantum Dots

QDs are well known for their versatile optical properties and currently, extensive research is focused on new methods to exploit these particles. One unique property for biochemical applications is the fact that QDs often luminesce brightly in the visible area of the spectrum when exposed to UV. Therefore, these particles could be used for multimodality imaging purposes. However, their applicability so far has been very limited due to their composition of toxic metals.

In 2008 Ducongé *et al.* [15] described a novel method for the ^18^F-labeling of core/shell QDs. The CdSe/CdZnS QDs are well known for their luminescent properties [25,26] and the authors described the use of their labeled QDs for PET and optical whole body imaging. After preparation, the QDs were encapsulated with polyethylene glycol (PEG-phospholipid micelles) and this coating was further functionalized with thiol groups. To these functionalities a coupling *via* a maleimido-based prosthetic group was performed, which was recently developed for the ^18^F-labeling of peptides. The maleimido reagent 1-[3(2-^18^F]fluoropyridin-3-xyloxy)propyl]pyrrole-2,5-dione ([^18^F]FPyME) [27] has been synthesized within 110 min in 20% yield (not decay corrected). The coupling of the QDs was performed by adding QDs in phosphate buffered saline (PBS) to the dried ^18^F-labeled maleimido reagent and subsequent vortexing for 15 min. The QDs were purified by gel filtration on a NAP-10 G25 sephadex cartridge, which yielded a 1.5 mL solution of ^18^F-labeled QDs. The total synthesis took 145 min and a activity concentration of 555–1110 MBq/mL was obtained when the reaction was started with 37 GBq of [^18^F]fluoride. The *in vivo* experiments showed a long blood circulation time of the QDs with a plasma half-life of 2 h and only small amounts of radioactivity in urine. In comparison to non-PEG-coated QDs, the uptake in liver and spleen could significantly be reduced. In *ex vivo* studies, no cadmium was detected in urine, indicating that the small amount of radiation found in the urine correspond to a low-molecular-weight degradation product under the renal threshold, implying that the QDs are not cleared from the body via the renal pathway.

### 2.2. ^18^F-Labeled Nanoparticles

NPs exist in a wide variety of sizes and materials, which is advantageous compared to the QDs. They can also be functionalized with different organic groups. These groups can be used to extend circulation time (e.g., PEG) or to conjugate a targeting moiety or a labeling agent. It is further possible to dope NPs with rare earth elements, which are known for their optical and magnetic properties, allowing the particles to luminesce or be used in MRI. Modification of these NPs allows them to serve as multimodality imaging agents.

In 2011 Liu *et al.* [16] developed ^18^F-labeled rare earth containing NPs. As a basis for their NPs Liu *et al*. used NaYF_4_, the particles were surface-modified with Gd^3+^ by cation exchange for Y^3+^ for use in MRI and different rare earth elements (Yb, Er) for use in luminescence studies. [^18^F]Fluoride was incorporated into the NPs through interactions with the rare earth ions in an aqueous [^18^F]fluoride (170 MBq) solution under sonication for 10 min. The NPs were separated by centrifugation and washed with water (3 times). Radio-TLC indicated an excellent labeling yield of 92%. Surface-modification of the NPs was carried out by coordination of carboxylic acid functions of different (bio)molecules to the surface of the NP. Thus, folic acid for active targeting was bound to the NP as well as oleic acid and aminocaproic acid. *In vitro* cell experiments demonstrated low cytotoxicity, *in vivo* studies showed a high uptake almost exclusively in the liver (80.9% ID/g) and spleen (36.6% ID/g) already after 10 min showing a decrease in the liver (53.5% ID/g) and further increase in the spleen (89.9% ID/g) 2 h p.i.. The rapid accumulation in spleen and liver was confirmed by MRI and optical imaging studies, furthermore, MRI studies showed a significant contrast enhancement due to the presence of the NPs. Considering the simple labeling method by just mixing [^18^F]fluoride with metallic (rare earth) NPs, the *in vivo*-stability is remarkable. An early, but stable bone uptake of ~13% ID/g revealed an initial loss of [^18^F]fluoride from the particles. 

Guerrero *et al.* [17] synthesized and studied ^18^F-labeled gold-peptide conjugates. Gold NPs were conjugated to an amphipathic peptide CLPFFD, which showed in a previous study the ability to remove *β*-amyloid aggregates, which are involved in Alzheimer’s disease [28,29]. *N*-Succinimidyl-4-[^18^F]fluorobenzoate ([^18^F]SFB) was prepared as prosthetic group and used to radiolabel the NPs. [^18^F]SFB was synthesized starting with drying and activating of ^18^F in the presence of the amino polyether 1,10-diaza-4,7,13,16,21,24-hexaoxabicyclo[8.8.8]hexacosane (Kryptofix^©^ K2.2.2) and K_2_CO_3_. After azeotropic drying, the precursor 4-(*tert*-butoxycarbonylmethyl)phenyl trimethylamonium trifluoromethanesulfonate, dissolved in acetonitrile, was added and heated to 90 °C for 10 min. Final deprotection was facilitated by 1M HCl (100 °C, 5 min). [^18^F]SFB was purified on a C18-HELA cartridge. The eluate was treated with 25% methanolic tetramethylammonium hydroxide solution. After drying, the activated ester was formed by addition of *N,N,N’,N,’*-tetramethyl-*O*-(*N*-succinimidyl)uronium tetrafluoroborate (TSTU). The product was purified by semipreparative HPLC with a RCY of 37%, a radiochemical purity (RCP) of ≥99% and specific activity of 110 ± 15 GBq/μmol. The labeling of the NPs was performed by first drying the NPs by centrifugation and re-dissolving them in DMSO/sodium citrate, followed by the addition of the NPs to 4.44 ± 1.11 GBq of dry [^18^F]SFB. This mixture was stirred for 1 h before sodium citrate was added and centrifugation of the whole mixture was performed. Subsequent washing was repeated until no more radioactivity was released. The residual solid was re-dissolved in a mixture of sodium citrate and Tween80. Guerrero *et al*. reported for the final product an average of 27 ^18^F atoms per NP (derived from calculation from the radioactivity-to-mass-ratio) with a labeling yield of 0.8% ± 0.3%. During *in vivo* studies it was observed that 120 min p.i. a considerable amount of the NPs was trapped by the reticuloendothelial system (RES) in the spleen. Furthermore, a fast renal clearance was observed leading to a rapid blood concentration drop during the first minutes p.i.

Ceria based NPs carrying an amino functionality were studied by Rojas *et al.* [18] Ceria-NPs were surface modified by silylation with 3-(aminopropyl)triethoxysilane to enable subsequent coupling to [^18^F]SFB. The [^18^F]SFB was prepared using the standard method already used by Guerrero *et al.*, with almost identical results, a RCY of 37% ± 5%, a RCP of 98% and a specific activity of 102 ± 7 GBq/μmol. The ceria-NPs (2.0 mg ± 0.3 mg) were labeled by stirring them for 1 h in a DMSO (150 μL) phosphate buffer (100 μL; pH = 7.4) mixture with ~2.6 GBq of [^18^F]SFB. After the reaction additional phosphate buffer was added and the mixture was centrifuged. The solid was then washed twice with phosphate buffer by centrifugation and the supernatant was checked for radioactivity, leading to a RCY of 18%. After suspension of the NPs in phosphate buffer, they were injected intravenously in Sprague-Dawley rats. High uptake values in liver, spleen and lungs were immediately observed after administration. At 2 h p.i., almost all particles were cleared from the blood. The remaining particles in the blood pool were further cleared *via* renal excretion. Only a very limited uptake in the brain was observed.

Sarparante *et al.* [19] functionalized porous silica NPs with hydrophobin as a self-assembled protein coating to study the *in vitro* and *in vivo* behavior using PET. Thermally hydrocarbonized porous silicon (THCPSi) NPs were synthesized and labeled with ^18^F. Labeling was facilitated *via* a direct ^18^F-fluorination using the Si-F-bond formation as driving force. Dry cryptate complex (K.2.2.2./K_2_CO_3_-system) was dissolved in anhydrous DMF containing 4% (*v/v*) acetic acid. The solution was added to 1 mg of the NPs suspended in DMF and the mixture was heated to 120 °C for 10 min. The particles were separated by centrifugation and repeatedly washed in ethanol and water using sonication. Finally, the particles were suspended in ethanol. The radiolabeled particles were covered with HBFII (which is a fungal protein class II hydrophobin). In *in vivo* studies (rat model, RAW 264.7 macrophages and HepG2 liver cells), the ^18^F-HFBII-THCPSi NPs accumulated mainly in liver and spleen. The HFBII coating improved the pharmacokinetics and biodistribution compared to the uncoated variant ^18^F-THCPSi. However, a slow HFBII-leaching from the coated NPs reduced the benefit of the HFBII coating and restored a fast renal clearance of the NPs.

An elegant direct way of labeling Al_2_O_3_-NPs with ^18^F was developed by Pérez-Campaña *et al.* [20]. Al_2_O_3_-NPs were directly activated in a cyclotron via the nuclear reaction ^18^O(p,n)^18^F. [^18^O]Al_2_O_3_-NPs were obtained by reacting AlCl_3_ in ammonia and ^18^O-enriched (100%) water. A short irradiation time (beam time 6 min, current 5 µA) yielded high amounts of radioactivity (2.3 ± 0.2 MBq/mg, saturation yield = 225.45 MBq/µA) of which 71% ± 4% could be attributed to ^18^F, the residue being assigned to ^13^N, which is always formed during ^18^F-production in a cyclotron *via* the ^16^O(p,α)^13^N reaction. Due to the much shorter half-life of ^13^N (9.7 min) the samples could be left for the ^13^N to decay without a significant loss of ^18^F. A second irradiation experiment was carried out with pure Al_2_O_3_, after irradiation only trace amounts of ^18^F could be detected as was expected from the natural abundance of ^18^O (0.201%), showing that there are no side reactions on the alumina. PET scans were carried out in dynamic mode and showed a renal excretion of the NPs, while a long retention of radioactivity in the heart indicated a long blood circulation half-life. PET imaging further revealed a minimal uptake in bones, suggesting that ^18^F might slowly leak from the NPs. Stability tests in rat serum at 37 °C, however, confirmed a stable ^18^F-label on the NPs with no degradation over a period of 8 h.

**Figure 3:**
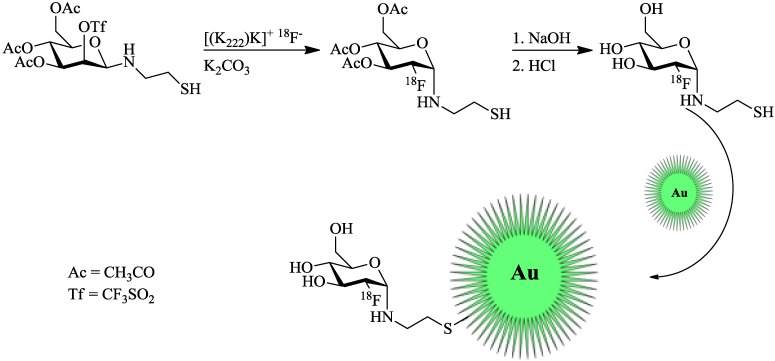
Radiolabeling of thiol-functionalized Au-NPs using a maleimido-[^18^F]FDG. [^18^F]FDG was produced in accordance with the standard protocol [21].

Unak *et al.* [21] worked on radiolabeled gold NPs. However, they only tested them *in vitro* in cancer cell cultures (MCF7, human breast adenocarcinoma cell line). Gold NPs were labeled with modified [^18^F]FDG. The cysteamine derivative [^18^F]FDG-CA was synthesized from a mannose-triflate cysteamine (Man-CA) precursor. Man-CA was produced according to standard methods, which entailed the addition of mannose-triflate to a mixture of cysteamine and NaCNBH_3_. Direct ^18^F-labeling of Man-CA was performed with [^18^F]fluoride (7.4 GBq) in the K2.2.2./K_2_CO_3_ system and DMF at 90 °C for 20 min. Purification was facilitated by elution from different ion exchange columns and a C18 cartridge. Labeling and synthesis of the gold NPs was carried out by mixing the previous prepared [^18^F]FDG-CA with 20 mM HAuCl_4_ solution and the addition of a NaBH_4_ solution (Figure 3). The mixture was stirred at 60 °C for 2 h, giving the desired ^18^F-labeled gold NPs. anti-metadherin (anti-MTDH) was conjugated to the particles for an active targeting of metadherin, which is overexpressed in many breast cancer cell-lines. Coupling of anti-MTDH was done by using 1,1'-carbonyldiimidazole as a reagent, involving multiple shaking steps, whereof two were 45 min each. The incorporation of these NPs into MCF7 breast cancer cells was higher than that of free ^18^F (2%) or [^18^F]FDG-CA alone (3%), 30% and 33% respectively for NPs without and with anti-MTDH. Additional apoptosis studies showed the expected decrease *in* the apoptotic effect for the NPs (20%), when compared to [^18^F]FDG-CA with 30% apoptotic effect, which is known for cysteamine in combination with radiation.

**Figure 4:**
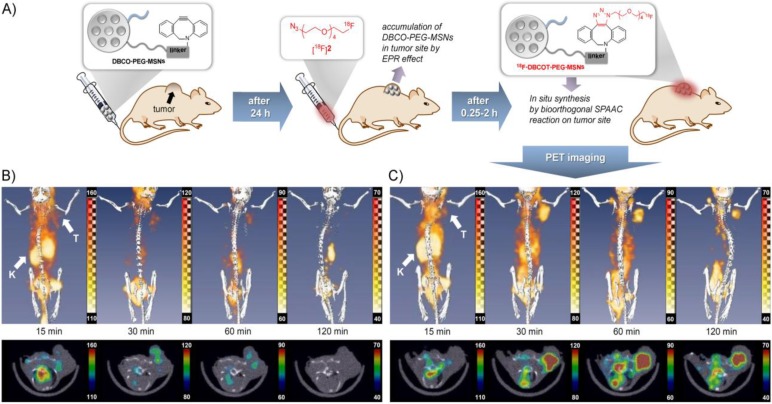
(**A**) Pre-targeting/labeling protocol for *in vivo* click reaction. (**B**) 3D PET images (upper row) and transversal slides (lower row) of a U87 MG tumor-bearing mouse injected with ω-[^18^F]fluoro-pentaethylene glycolic azide without pretargeting. (**C**) 3D PET images (**upper row**) and transversal slides (**lower row**) of a U87 MG tumor-bearing mouse injected with ω-[^18^F]fluoro-pentaethylene glycolic azide with pretargeting using DBCO-PEG-NPs. Reprinted with permission from S.B. Lee *et al.* [22]; Copyright 2013 John Wiley and Sons.

Recently, Lee *et al.* [22] described a bioorthogonal labeling strategy where they applied a copper-free click reaction *in vivo* for ^18^F-(pre)labeling of NPs (Figure 4). The radiotracer used in this experiment consisted of ω-[^18^F]fluoropentaethylene glycolic azide with a specific activity of 42 GBq/μmol. NPs where modified with azadibenzocyclooctyne (DBCO) and PEG. The reaction was first tested in PBS at 36.7 °C to test if the copper-free click reaction has a chance to work *in vivo*, this gave almost quantitative yields within 15–20 min. The modified NPs were injected and allowed 24 h to accumulate in tumors via the EPR effect. After 24 h, the radiotracer was injected and PET images were acquired. A control group was measured, which was given only the radiolabeled product and not the NPs. The comparison between the two groups showed similar uptake in all tissues except for the tumor, whereas the pre-targeted animals showed a much higher tumor uptake than the non-targeted animals which confirms that the copper-free click reaction proceeds efficiently *in vivo*. Furthermore, it was found that increasing the amount of radiolabeled compound also increased the tumor uptake and thereby the signal-to-noise ratio.

### 2.3. ^18^F-Labeled Polymers

Polymers can appear in many different compositions and architectures. The fact that polymers are derived from organic materials means that they can easily be modified using organic chemistry techniques and that ^18^F is covalently bound to the molecule, limiting the possibility of leaching. Polymers can be modified to carry targeting agents and/or their architecture can be varied. Some of the known polymers are biodegradable *in vivo* with no toxicity at all. Furthermore, several different polymer/drug conjugates are known, e.g., pHPMA-doxorubicin [30], and about twelve have been approved for the market and about fifteen additional conjugates have entered clinical trials to treat different diseases [31].

The polymers discussed in the following section were all labeled using the prosthetic group radiolabeling method for HPMA-based polymers described by Herth *et al.* in 2009 [4]. Generally, for the radiolabeling ^18^F was first dried and activatedas cryptate complex (K2.2.2/K_2_CO_3_-system). The precursor ethylene-1,2-ditosylate was added in acetonitrile and heated for 3 min in a sealed vial to complete the ^18^F-labeling. Purification from crude mixture was facilitated by HPLC (Lichrosphere RP18-EC5, acetonitrile/water). The HPLC-fraction containing the 2-[^18^F]fluoroethyl-1-tosylate ([^18^F]FETos) was diluted with water (1:4 HPLC fraction/water) and loaded on a C18-Sep-Pak cartridge (Waters, Milford, MA, USA), dried by a nitrogen stream and eluted with DMSO, ready for further coupling reactions. The whole preparation takes around 40 min with a RCY from 60% to 80%. Labeling of the polymer was typically done by dissolving about 3 mg of polymer in DMSO followed by the addition of 1 μL of 5N NaOH solution and addition to the [^18^F]FETos solution. The coupling reaction was performed at temperatures between 80 and 150 °C for 20 min. Radio-size exclusion chromatography (SEC) was used to separate the smaller compounds from the labeled polymer. The SEC is performed with physiological saline solution and hence, the collected polymer fraction is directly available for *in vivo* studies.

Herth and co-workers described the labeling of an HPMA-polymer by using [^18^F]FETos, which was coupled to the hydroxyl group of a tyramine moiety which was previously incorporated in the polymer during the polymer analogous reaction [4]. Several polymers of different molecular weights were labeled with ^18^F and a systematic study was done to find the optimal ^18^F-labeling conditions. Best results were obtained using 120 °C for 10 min in DMSO. Moreover, at 60 °C 20% RCY could be obtained within 20 min leading the authors to conclude that labeling at room temperature would be possible. The authors further reported that the compound was cleared from the body predominantly via the urine and that initially after injection some metabolism was visible.

In 2011 a follow-up was made to this study by Allmeroth and Moderegger *et al*. [23] where different polymers were synthesized and ^18^F-labeled. The polymers were either HPMA homopolymers or HPMA-*ran*-LMA (LMA = lauryl methacrylate) copolymers. Different molecular weight (M_W_) polymers were synthesized with sizes ranging from 12,000 to 130,000 g/mol. The obtained RCY in this study varied and a trend was noticeable that the RCY decreased with increasing M_W_, the RCY also decreased when HPMA-*ran*-LMA copolymer was used instead of the HPMA-homopolymer. During *in vivo* studies, a clear difference was noticeable between the high and low M_W_ polymers. Expectedly, the low M_W_ polymers tended to renal excretion with 12% ID/g in kidneys while for the high M_W_ polymers this was reduced to 6.4% ID/g. The authors found a difference in circulation time between the homo- and *ran*-LMA-co-polymer with a similar M_W_. The HPMA-*ran*-LMA copolymer had a much longer blood circulation time with around 30% ID/g for the low M_W_ and 60% ID/g for the high M_W_ polymer still present in the blood at 2 h p.i.

HPMA-*b*-LMA block-copolymers were employed in 2013 [24] to study the effect of different grades of PEGylation. This work was specifically focused on trying to avoid aggregate formation and reducing protein interactions by coating the polymer with amino-functionalized PEG_2000_ fragments. ^18^F-Labeling of the polymers was again carried out with [^18^F]FETos, however, this time Cs_2_CO_3_ was used as base instead of K2.2.2/K_2_CO_3_. The authors came to the conclusion that the introduction of the PEG_2000_ as well as the degree of PEGylation has a great influence on the pharmacokinetics of the polymer. Thus, the polymer with only a small percentage of PEGylation still showed a fast clearance via the liver and a short blood circulation time. An increase in the PEGylation grade correlated with an increase in the blood circulation time and an increased tumor uptake in the Walker-256 carcinoma (rat model).

^18^F-Labeling of NPs and polymers is just at the very beginning, but the first examples have already shown their potential in both the suitability of the radiochemistry and the benefit of the pharmacological information from PET imaging. There is still a broad range of methods for the ^18^F-introduction to be explored for their applicability to macromolecular drug delivery systems and further studies and research will quickly follow.

In the next section, the radiolabeling for NPs and polymers and their preliminary preclinical evaluation using the metallic PET nuclides ^68^Ga and ^64^Cu is summarized and discussed.

## 3. Labeling of Polymers and Nanoparticles with ^64^Cu and ^68^Ga

**Figure 5:**
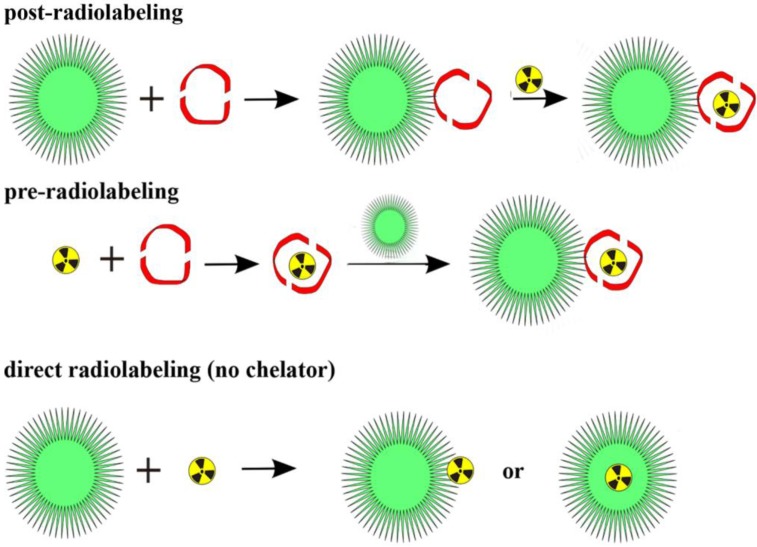
Three general radiolabeling approaches using metallic radionuclides and nanoparticles.

There are only a few radiometals suitable for PET, including ^64^Cu, ^68^Ga and ^89^Zr, which are attached to (polymeric and nanodimensional) molecules via BFCs. Thus, the chemistry is quite different from that of covalently bound radionuclides. There are two main roots to label a polymeric or nanodimensional system (Figure 5). Either the label is attached to the whole polymer/particle, which has been synthesized in advance, or one compound (in the case of metals this is a BFC) is labeled first and subsequently the polymer/particle is formed. Whether to use the so called pre- or postradiolabeling [32,33] is dependent on several factors, above all the half-life of the chosen radionuclide. In this section, the approaches in ^64^Cu and ^68^Ga-(nano)chemistry are discussed.

### 3.1. Radiolabeling with ^64^Cu

Copper-64 with a half-life of 12.7 h, its a positron energy E_β+,max_ of 655 keV and an β^+^ intensity of 17% [34] is a frequently used PET nuclide which is available via different production routes. The most common one is the ^64^Ni(p,n)^64^Cu nuclear reaction providing good yields of up to 241MBq/µAh [33,34,35]. 

As a radiometal, copper-64 requires a BFC for attaching it to biomolecules and forming stable complexes. The most popular one is DOTA or derivatives of this macrocycle. Originally, DOTA was designed for lanthanides (e.g., Gd^3+^), but it can be used for a wide range of (radio)metals as well. Since DOTA has four carboxylic functions on the side-chains of the macrocycle bearing four nitrogens, leading to a deformed octahedral complexation of the Cu^2+^-ion, which is preferred due to the Jahn-Teller effect [36] thereby leaving two of the acidic functions “free”. Thus, one is available for the coupling to a molecule (in our case NPs or polymers) and the other one allows further derivatization or acts as additional hydrophilic group. Additional macrocyclic chelators are TETA (1,4,8,11-tetraazacyclotetradecane-*N,N',N'',N'''*-tetraacetic acid) and NOTA or derivatives of those. 

The general ^64^Cu-labeling conditions in literature are very similar: Since the product of the bombardment at the accelerator (after target dissolving and work-up) in every case is CuCl_2_ in hydrochloric acid, this species is transferred into the copper(II)acetate by adding ammonium acetate buffer. Subsequently, the solution of the polymer/particle is added and the mixture is heated for 30–240 min [32,37] to 40–43 °C [32,37,38,39,40,41,42] or to 80–95 °C [43,44,45,46,47]. The pH-value varies from nearly neutral (7.4 [40]) to slightly acidic 5.5 [47,48,49,50,51].

In the same manner, purification methods of crude labeling mixtures are quite similar. The excess of copper is removed by either adding another chelating agent (in most cases DTPA [37,40,41,42,43,46,50], but also EDTA is used [44,48,50]), bySEC (with PD10 [38,40] or by a filter centrifugation [47,49,50,51]).

Table 2 gives an overview on the different approaches in radiolabeling NPs and polymers with ^64^Cu and ^68^Ga.

**Table 2:** An overview of different approaches of ^64^Cu-labeled nanoparticles and polymers and important parameters. (n.d. = no data, RCY = radiochemical yield, RCP = radiochemical purity, PAA-PMA = polyacrylic acid – polymethacrylic acid, PS-PAA = polystyrene – polyacrylic acid, PMMA = polymethylmethacrylic acid, PMASI = polymethacryloxy-succinimide).

Nanostructure/ system	Material	Size [nm]	Chelator	Labeling time	T [°C]	pH	RCY (RCP) [%]	Specific activity	Ref.
organic polymer (star or arm)	mPEG (metoxy-terminated PEG)	25–70	DOTA	n.d.	n.d.	n.d.	n.d. (≥95%)	≥3700 kBq/µg	[52]
core-shell arm or star copolymers	PEG, *N,N*-dimethylacrylamide, *N*-acryloxysuccinimide	5–70	DOTA	1 h	80	n.d.	n.d. (≥95%)	185-370 GBq/mg	[43]
organic polymers	PAA-PMA, PEG (folic acid)	20	TETA	2.5 h	43	7.4	15%–20% (95%)	n.d.	[40]
inorganic QDs	silicone	15	DOTA	n.d.	n.d.	5.5	78%	n.d.	[48]
inorganic NPs	iron oxide	30	DOTA	1 h	40	6.5	n.d.	n.d.	[39]
organic polymers	PS-PAA	13–47	DOTA	2 h	43	n.d.	n.d.	n.d.	[42]
organic polymers	poly( *t*-butyl acrylate), PEG, methyl acrylate, styrene	18–37	TETA	2–4 h	43	n.d.	n.d.	n.d.	[37]
organic polymers	PMMA, PMASI, PEG	10–20	DOTA	1 h	80	n.d.	n.d. (≥95%)	0.4–0.8 MBq/µg	[46]
organic polymer	CANF (C-atrial natriuretic factor) comb	20, 22	DOTA	1 h	80	n.d.	60:5% ± 7.3%	n.d	[45]
organic polymer	CANF (C-atrial natriuretic factor) comb	n.d.	DOTA	1 h	80	n.d.	n.d.	n.d.	[44]
inorganic NPs	iron oxide	68	NOTA	40 min	40	6.5	n.d.	n.d.	[40]
inorganic NPs	silicone	77	DOTA	1 h		5.5	n.d.	n.d.	[49]
organic polymers	glycol chitosan	300	DOTA	30 min	40	n.d.	≥98%	11 MBq/mg	[32]
QDs	CdSe	12; 21	DOTA	1 h	37	5.5	≥95%	≥37 GBq/µmol	[51]
inorganic NP	dextranated iron oxide	20	DTPA	25 min	95	5.5	n.d.	370 MBq/mg Fe	[47]
QDs	CdSe and InAs	2; 12	DOTA	1 h	37	0.1 5.5	n.d.	n.d.	[53]
inorganic NPs	iron oxide	20	DOTA	1 h	37	5.5	94% (≥95%)	2–4 GBq/mmol	[40,50]

As mentioned before, two general radiolabeling approaches are available, pre- and postradiolabeling. Several examples are described and discussed below.

#### 3.1.1. Post-Radiolabeling

Most of the labeling procedures described here, are “post-radiolabeling” processes [32,33], meaning that the polymeric or nanoparticular system is formed first, a chelator is coupled to this system and in the last step the radiolabel is introduced. This procedure has been applied to various nanodimensional structures, for organic polymers as well as for inorganic NPs.

##### ^64^Cu-Labeled Organic Polymers

Pressly and coworkers attached DOTA to a C-type atrial natriuretic factor (CANF) functionalized Comb-copolymer for targeting the natriuretic peptide clearance receptor in prostate cancer [44,45]. After the self-assembly of the DOTA-CANF-Comb-copolymer to a particle, ^64^Cu-radiolabeling was done by adding approximately 5 pmol of the readily prepared particles to a solution of 185 MBq ^64^Cu at pH 5.5 at 80 °C. Subsequently, both purification methods were applied, EDTA-challenge as well as a desalting column. The ^64^Cu-labeled CANF-Comb-copolymers were tested in CWR22 tumor mice and compared with a non-targeted version, ^64^Cu-Comb-copolymer. The targeted version showed significantly higher tumor uptake of ^~^9% ID/g (24 h) than ^64^Cu-Comb-copolymer (~3% ID/g, 24 h). Moreover, the uptake of the ^64^Cu-CANF-Comb-copolymers decreased in all non-targeted tissues and organs over time (1-24 h), while the tumor accumulation increased over time from ~4% to ~9% ID/g. Under blockade conditions (100-fold excess of inactiveCANF-Comb-copolymer) the tumor uptake was significantly reduced, and thus CANF-specificity was confirmed [44].

Welch *et al.* have chosen a similar root, by coupling DOTA to a polymer via amide formation, and performed radiolabeling with ^64^Cu after self-assembling [52]. It was demonstrated that the shape of the polymer (*star-* or *arm-shaped*) has a stronger influence on its biodistribution in BLAB/C mice than the size or molecular weight, respectively. Thus, in contrast to the arm polymer, the star polymer was found to have an extended blood circulation time. Additionally, the arm polymer showed a greater uptake in liver and spleen than the star polymer [52].

Rossin *et al.* combined the passive targeting that NPs automatically undergo with active targeting by attaching folic acid to their shell-cross-linked micelles. Thus, they could ratify the EPR-effect, but at the same time they could not see a clear difference between the folate-conjugated tracer and the polymer without targeting-vector [40]. TETA was used as chelating agent and conjugated to shell-cross-linked nanoparticles (SCKs) composed from an amphiphilic block-copolymer. The SCKs were mixed with 185 MBq ^64^Cu and reacted at slightly increased temperatures for 2.5 h, before free copper was removed by a DTPA-challenge, giving a RCP of ≥95%. Although there was no obvious difference between the targeted and the non-targeted micelles in tumor uptake (human KB cells, female nu/nu mice), the liver uptake increased due to the functionalization with folic acid [40].

##### ^64^Cu-Labeled Inorganic Nanoparticles

In most cases, the inorganic substances that are described are silicon-based or iron-based NPs. In addition, some groups exploit the physical properties of the metals, such as their magnetic behavior or their suitability as MRI or CT contrast agents. Such NPs show potential as dual modality imaging agents. A combination of a morphological imaging technique (MRI/CT) and PET as a functional imaging technique is one of the most potent imaging systems. 

Tu *et al.* coated manganese-doped silicon QDs with dextrane and coupled a DO3A-derivative as chelator. ^64^Cu-Radiolabeling was facilitated in acetate buffer (pH 5.5), followed by an EDTA-challenge. After centrifuge filtration, a RCY of 78% was achieved. *In vivo* PET images show that the QDs were retained in the bladder and liver for over 1 h p.i. and can still be found in the liver at 48 h p.i. In general, a rapid blood clearance was observed (<2.5% ID/g 10 min p.i. in the blood) [48]. Originally, DOTA-NHS-ester was applied as chelator, but the activated ester did not couple to the particles’ surface. The authors assumed that the side chain was slightly too short and designed a DO3A-derivative with a propylamine chain. As a result, the coupling worked in satisfying yields and enabled radiolabeling [48].

Huang *et al.* loaded mesoporous silica nanoprobes with a near infrared (NIR)-dye, and labeled the nanostructures with two different metal ions, which were Gd^3+^ (T_1_-contrast agent in MRI) and ^64^Cu^2+^ for PET imaging, respectively. As a chelating agent DOTA was employed on the one hand, and on the other hand the authors exploited the fact that both the copper and the gadolinium embed into the surface pores. The labeling conditions were 1 h of constant shaking at a pH of 5.5 and 40 °C. Stability tests showed that the procedure delivers a highly stable radiotracer, which exhibits excellent uptakes in the sentinel lymph node (SLN), which could be demonstrated in PET imaging using BALB/C mice carrying 4T1 tumors [49,54].

Non-iron and non-silica-based inorganic particles have been radiolabeled by Schipper *et al.* to investigate the biodistribution in living mice of CdSe-QDs, which carry DOTA at their surfaces. They labeled the particles at a pH of 5.5, 37 °C and for 1 h and gained excellent RCYs of ≥95% and quite high specific activities of ≥37 GBq/µmol. *In vivo* microPET images and biodistribution data showed a delayed uptake in the organs of the RES (liver and spleen) if the QDs are coated with PEG or peptide(s). Additionally, the group could show that hydrodynamic diameters of their QDs (12 nm and 21 nm) had no influence on the biodistribution [51,53]. 

#### 3.1.2. Pre-Radiolabeling

Recently, Dong-Eun Lee *et al.* followed a very interesting approach to radiolabel glycol chitosan nanoparticles (CNPs) [32]. Copper-free click chemistry was applied to attach a ^64^Cu-radiolabeled alkyne complex to azide-functionalized CNPs *in vivo*. The general labeling procedure was (due to DOTA as chelator) 30 min at 40 °C, where a DOTA-DBCO-conjugate was labeled with excellent RCYs of more than 98% within 30 min at 40 °C. PET imaging in SCC/-tumor-bearing mice showed promising results with an excellent tumor-to-background contrast. Furthermore, a significant uptake in liver and kidneys was observed.

### 3.2. Radiolabeling with ^68^Ga

The amount of reports about ^68^Ga-labeled polymers and NPs is much smaller than for ^64^Cu. Table 3 gives an overview. Gallium-68 decays with a half-life of 67.71 min under β^+^-decay into stable zinc-68. It has 89% positron branching accompanied by 3.22% γ-emission [55]. Its positron energy E_β+,mean_ of 740 keV [55] is ideal for PET imaging and provides a high spatial resolution.

**Table 3:** An overview of the different approaches of ^68^Ga-labeled nanoparticles and polymers and crucial parameters. (n.d. = no data, RCY = radiochemical yield, RCP = radiochemical purity, CAN = cerium-ammonium-nitrate).

Nanostructure/ system	Material	Size [nm]	Chelator	Labeling time [min]	T [°C]	pH	RCY (RCP) [%]	Specific activity	Ref.
organic nanogels	PEG	250–270	NODAGA	15	RT	4.5	≥99%	≥1500 GBq/g	[56]
inorganic NP	iron oxide, oleanic acid	60	NOTA	20	RT	5.0–5.5	n.d.	n.d.	[57]
inorganic NP	γ-Fe_2_O_3_, CAN; PEG-coat	44–55	NODAGA	30	60	3.5	84% ± 6%	n.d.	[11]
superparamagnetic NPs	iron oxide amino-silane coated	100	none	20	70	n.d.	(≥95%)	358 MBq/nmol	[58]
organic polymer	poly-glycidyl-methacrylate(poly-2,3-epoxy-propylmethacrylate)	144	none	15	82–60	n.d.	n.d.	0.2 MBq/mg	[59]

In contrast to copper-64, gallium-68 is a generator-produced nuclide. It is the daughter of germanium-68 which has a half-life of 270.8 d and decays under electron capture into gallium-68 [55]. Thus, as a major advantage no on-site cyclotron is required and the costs for nuclide production are much lower.

Gallium, as well as copper, requires a chelating agent, which can be DOTA (frequently used for copper), but also NOTA which is a highly potent chelator for the smaller gallium(III)-cation. So far, only NOTA and its derivatives have been used to radiolabel organic and inorganic nanodimensional systems with gallium-68.

Due to the fact that the chelator is the same (or at least a derivative of NOTA) the quite different labeling methods are surprising. Sing *et al.* nearly had quantitative RCYs by labeling nanogels after 15 min at room temperature [56], whereas Locatelli and coworkers used much higher temperatures and twice as much time and did not reach more than 90% RCY [11]. Both used NODAGA as chelating system. Noteworthy, the labeled structures are of fundamental difference. Locatelli *et al.* described inorganic NPs composed of γ-Fe_3_O_4_ and cerium ammonium nitrate, whereas Singh *et al.* investigated (organic) polymeric nanogels.

Locatelli *et al.* also performed PET imaging studies with male Sprague Dawley rats to find out that the uptake in liver, spleen and lungs is quite high. A high uptake and long retention in the heart indicates a long blood circulation time [11].

In contrast to the radiocopper chemistry, in the radiogallium labeling chemistry methods like DTPA-challenge are rather exotic. Purification methods and procedures for ^68^Ga-radiotracers are commonly based on SPE (solid phase extraction) cartridges as well as SEC. Due to the time-consuming procedure, the latter is unfavorable. However, Singh *et al.* employed PD10-columns to separate the ^68^Ga-labeled product from the crude reaction mixture, and Locatelli *et al.* used ultracentrifugation for purification [11,56].

Kim *et al.* applied very mild labeling conditions. By adjusting the pH to 5.0–5.5 [57] they are at the upper limit of the suitable pH-range for ^68^Ga-labeling reactions. Oleanic acid conjugated iron oxide NPs (IONPs) (carrying again NOTA) were successfully radiolabeled within 20 min at room temperature. As a result, a potent dual imaging agent for MRI and PET was prepared. They show very nicely a significant uptake in the tumor for both imaging systems, MRI and PET [57].

A quite interesting work was reported by Stelter *et al.,* where they did not use any chelating agent at all. They simply utilized the fact that primary amines can form stable complexes with gallium(III) and coated their particles with aminosilanes. For radiolabeling, 130 MBq ^68^Ga were added for 20 min at 70 °C. Afterwards a DTPA-challenge which is common in copper-radiochemistry was employed. Hence, they could on the one hand eliminate the surplus ^68^Ga and on the other hand ensure the stability of their compound against transchelation. They gained excellent RCPs (≥95%) and demonstrated feasibility in *in vivo* small animal PET studies in Wistar rats. The compound only accumulated in liver and spleen [58].

A similar approach, *i.e.*, without chelator, was published by Cartier *et al.* in 2007. They used EPMA-particles (poly-2,3-epoxypropylmethacrylate) and 300 MBq ^68^Ga for radiolabeling and started at temperatures of about 82 °C and let it drop to 60 °C during a period of 15 min. After purification via PD10, the specific activities were 0.2 MBq/mg lattice. Injection into Wistar rats and PET imaging showed that this compound accumulates in the liver 1 h p.i [59]. 

In gallium-chemistry, no pre-radiolabeling approach like in copper-chemistry has been published, yet. Of course, the much shorter half-life of the gallium-68 plays the predominant role, here. However, only the development of faster labeling methods and rapid coupling/conjugation chemistry would enable radiolabeling via the pre-radiolabeling approach for ^68^Ga. On the other hand, the existing labeling strategies enable the development of pre-targeting approaches based on ^68^Ga, which is particularly suitable for the application with NPs and polymers.

## 4. Labeling of Polymers and Nanoparticles with Other Positron Emitters

As already mentioned before, a broad variety of different radionuclides with a wide range of half-lives available for NPs and polymers is of paramount interest. Such a pool of nuclides is crucial to meet the imaging requirements for following a slow pharmacological process like the EPR effect with PET measurements over a longer period (*i.e.*, days). On the other hand, data about the initial biodistribution and pharmacokinetics have already proven a high significance for the applicability of a potential drug delivery system. Besides, the major radionuclides ^18^F, ^64^Cu and ^68^Ga, several other positron emitters have been applied for radiolabeling of NPs and polymers (Table 4). Remarkably, the corresponding half-lives are spread over a very wide range from minutes to weeks, and thus offer very interesting possibilities with a great flexibility.

**Table 4:** Further positron emitters used for radiolabeling of polymers and nanoparticles and their decay properties [13,60,61,62].

Positron Emitter	Half-life	Decay Properties (%)	β^+,max-energy^ [MeV]	Production Route	Daughter (T_½_)
^13^N	9.97 min	β^+^ (100)	1.19	^16^O(p,α)^13^N	^13^C (stable)
^11^C	20.4 min	β^+^ (99.8)/EC (0.2)/	0.96	^14^N(p,α)^11^C	^11^B (stable)
^86^Y	14.7 h	β^+^ (33)/EC (66)/γ	3.14	^86^Sr(p,n)^86^Y	^86^Sr (stable)
^76^Br	16.2 h	β^+^ (55)/EC (45)/γ	3.94	^76^Se(p,n)^76^Br	^76^Se (stable)
				^76^Se(d,2n)^76^Br	
^72^As	26.0 h	β^+^ (88)/EC (22)	3.33	^72^Se/^72^As (generator)	^72^Ge (stable)
^89^Zr	3.3 d	β^+^ (23)/EC (77)/γ	1.81	^89^Y(p,n)^89^Zr	^89^Y (stable)
^124^I	4.18 d	β^+^ (23)/EC (77)/γ	0.901	^124^Te(p,n)^124^I	^124^Te (stable)
^74^As	17.8 d	β^+^ (29)/β^-^ (61)	1.54	^74^Ge(p,n)^74^As	^74^Ge (stable)

The PET radionuclides discussed in this section are quite different in their half-lives, and they are similarly diverse in their (radio)chemistry. Consequently, the following examples show nicely how the choice of the radionuclide/-chemistry and nanostructures interact. Furthermore, the broad range of half-lives provides *in vivo* data from the first initial minutes up to several days. Table 5 gives an overview on the different combinations of radionuclides and NPs or polymers discussed in this section.

**Table 5:** An overview of radiolabeled nanoparticles and polymers using various positron emitters. (n.d. = no data, RCY = radiochemical yield (decay corrected), HPMA = *N*-(2-hydroxypropyl)-methacrylamide, PEO = polyethyleneoxide, h.d. = hydrodynamic radii).

Positron Emitter (T_½_)	Nanostructure/ System	Material	Size [nm]	Labeling Time [min]	T [°C]	RCY (RCP) [%]	Specific Activity	Ref.
^13^N (9.97 min)	nanoparticle	Al_2_O_3_ (alumina)	10–10,000	6 (beam time)	n.d.	1.9 MBq/mg	1.9 MBq/mg	[10]
^11^C (20.4 min)	nanoparticle	iron oxide-COOH	16	5 min (methylation)	125	0.3	n.d.	[63]
		iron oxide-NH_2_	16			2.3		
		silica-NH_2_	32			3.2		
		platinum-COOH	2.5			7.6		
^86^Y (14.7 h)	nanotube	carbon	47 ± 17	30	60	n.d. (90)	555 GBq/g	[64]
^76^Br (16.2 h)	polymer/dendri-mer	PEO	12 (h.r)	20	RT	n.d. (95)	190 kBq/µg	[65]
^72^As (26.0 h)	polymer	HPMA	n.d.	60–120	30–70	20–90	100 kBq/µmol	[12]
^74^As (17.8 d)								
^89^Zr (3.3 d)	nanoparticle						592 GBq/g	[66]
^124^I (4.18 d)	nanoparticle	iron oxide						[67]

**Figure 6:**
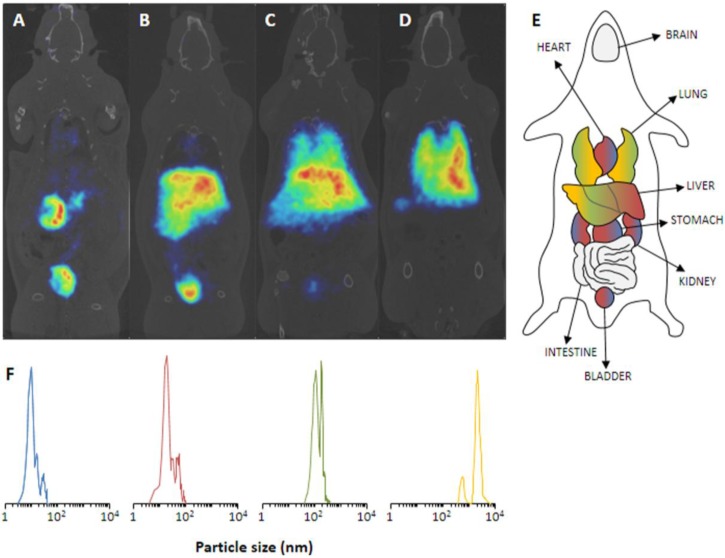
(**A**–**D**) PET images of ^13^N-nanoparticles of different size in Sprague-Dawley rats (60 min p.i.). (**A**) 10 nm, (**B**) 40 nm, (**C**) 150 nm, (**D**) 10 µm. (**E**) Schematic anatomical overview with localization of important organs. (**F**) The corresponding particles size distribution of the employed NPs. Reprinted with permission from Pérez-Campaña C. *et al.* [10]; Copyright 2013 American Chemical Society.

Pérez-Campaña *et al.* irradiated commercial Al_2_O_3_-NPs with a proton beam (16 MeV) and utilized the ^16^O(p,α)^13^N nuclear reaction to produce ^13^N-labeled NPs [10]. In the same manner, they already produced ^18^F-labeled NPs via the ^18^O(p,n)^18^F nuclear reaction on ^18^O-enriched Al_2_O_3_-NPs and demonstrated the feasibility of this approach [20]. Furthermore, the *in vivo* data (Sprague-Dawley rats) revealed an uptake plateau of the ^18^F-labeled NPs in organs after 1 h. Consequently, the cost-intensive ^18^O-enrichment of Al_2_O_3_-NPs can be avoided by facilitating the ^16^O(p,α)^13^N nuclear reaction and the shorter-lived radionuclide ^13^N (9.97 min). NPs of 10, 40, 150 nm and 10 µm were applied. The short half-life of 9.97 min enabled an *in vivo* PET imaging over a period of 68 min, thus, sufficient to track the pharmacokinetics and biodistribution of those NPs. The NPs were irradiated as solid in an aluminum capsule for 6 min with a 16 MeV proton beam of 5 µA. Subsequent suspension of the NPs into physiological saline and centrifugation already gave the injectable solution. *In vivo* PET studies in Sprague-Dawley rats show an expected biodistribution in strong dependence on the particles’ size (Figure 6). Accordingly, the smallest NPs of 10 nm exhibited an exclusively renal excretion with all radioactivity in kidneys and bladder/urine. With increasing size, the renal glomerular filtration cut-off was quickly reached and ^13^N-NPs of 40 nm still showed a renal fraction, but already a high uptake in liver. The tendency to lung accumulation increased with the larger particles, hence, 10 µm NPs were only found in lungs.

For the development of NPs for dual imaging (PET and MRI), Sharma *et al.* radiolabeled iron oxide NPs with the short-lived PET-radionuclide carbon-11 (T_½_ = 20.4) [63]. Additionally, they used silica and platinum NPs to establish the ^11^C-labeling procedure. The radiolabeling was based on the commonly used ^11^C-methylation via [^11^C]methyl iodide, which was produced on a commercial radiosynthesis module (Microlab, GE Medical Systems, Wauwatosa, WI, USA). ^11^C-methylation of the different NPs was enabled by surface modification with either amino functionalities or carboxylic acids, leading to the NPs (size) iron oxide-COOH (16 nm), iron oxide-NH2 (16 nm), silica-NH_2_ (32 nm) and platinum-COOH (2.5 nm). The ^11^C-methylation was facilitated by heating to 125 °C in DMF or DMSO for 5 min. RCY were varying from 0.3% to 7.6% in dependence of the NPs (see Table 5). The ^11^C-labeled NPs were purified only by washing and centrifugation. The low RCY for iron oxide NPs was assumed to be a result of particle agglomeration and low ligand density on the NP’s surface. However, the ^11^C-labeled NPs were stable (>95%) in plasma at 37 °C for at least 120 min. In proof of concept *in vivo* studies using the ^11^C-labeled magnetic iron oxide NPs in mice, dual imaging of the liver showed perfect matches for PET and MRI in fused images.

A PET radionuclide with a convenient half-life of 14.7 h is ^86^Y. McDevitt and co-workers used ^86^Y for the radiolabeling of carbon nanotubes (CNT) and studied the *in vivo* behavior in nude mice [64]. The surface of single walled carbon nanotubes was amino-functionalized and derivatized using *p*-NCS-DOTA for a stable thiourea formation with primary amino functions. The DOTA-CNT were mixed with a [^86^Y]YCl_3_-solution (296 MBq) for 30 min (60 °C, pH 5.5). The ^86^Y-DOTA-CNT was isolated via SEC (P6 resin (BioRad)). ^86^Y-DOTA-CNT was obtained in good radiochemical purities of ≥90% and with a specific activity of 555 GBq/g. The ^86^Y-labeled nanotubes were applied to healthy nude mice to study their *in vivo* behavior. *In vivo* PET imaging at 3 h p.i. showed major uptake in liver, kidneys and spleen, which was confirmed by *ex vivo* biodistribution studies 24 h p.i. giving 15.2% ± 1.5%, 5.96% ± 1.20% and 0.82% ± 0.04% ID/g, respectively. No further background activity was observed, only a minor accumulation in bones was detectable. Interestingly, the authors compared the *in vivo* data of two different administration protocols, intravenous and intraperitoneal injection. Both methods gave almost similar results in kidneys and spleen, only the liver uptake being higher after i.v. administration.

With an almost similar half-life (16.2 h), the radiohalogen ^76^Br was employed by Almutairi *et al.* for radiolabeling of a dendritic polymer [65]. Based on a core-shell architecture, an eight-branched polyethylene oxide (PEO) dendrimer was synthesized via NHS-esters. The dendrimer was functionalized with tyrosine moieties for a direct electrophilic radiobromination. Furthermore, the PEO-branches were coupled to cRDG peptides (~5 RDG/dendrimer) for active targeting the α_v_β_3_ intergin for angiogenesis imaging. ^76^Br-Radiobromination was facilitated in phosphate buffer (pH 7) in the presence of chloramine-T (CAT). After 20 min radiolabeling, purification via SEC (HiTrap cartridge, GE healthcare) gave the product in high radiochemical purities of ≥95%. In *in vitro* cell assays, the multivalent cRDG-dendrimer provided a 50fold increase in affinity (avidity) due to the multivalency with an IC_50_ value of 0.18 nM instead of 10.4 nM (mono-cRDG peptide). Similarly, a 6-fold increase in endocytosis was observed using the multivalent dendrimers. The ^76^Br-labeled dendrimers were further applied to mice with a hind limb ischemia. *In vivo* PET imaging 24 h p.i. showed high uptake in the diseased regions and *ex vivo* biodistribution studies 4 h, 24 h and 48 h p.i. indicated a relatively fast renal clearance, whereby a prolonged retention in kidneys was assigned to a specific binding/uptake of the RDG-groups.

**Figure 7:**
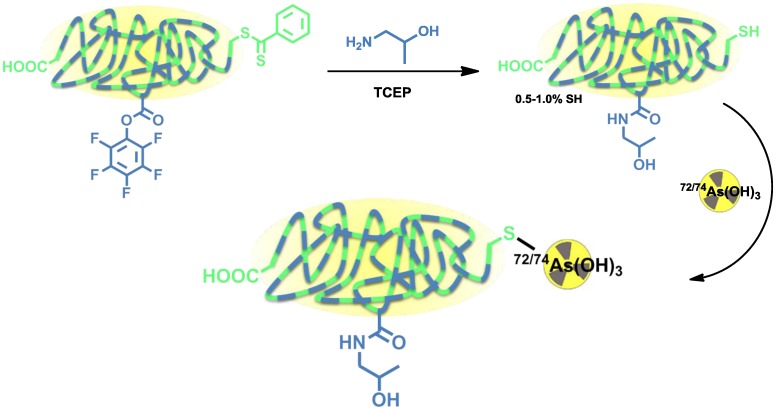
Thiol-functionalization of HPMA-based polymers and radiolabeling strategy for ^72/74^As-labeled HPMA-based polymers.

Herth *et al.* developed the radiolabeling of HPMA-based polymers for ^72/74^As (Figure 7) [12]. Radioarsenic was achieved from a proton (15 MeV) irradiation of a natural germanium target leading to 4 GBq ^72^As (30 µA beam current) and 400 MBq ^74^As (200 µA beam current), respectively. To enable the radiolabeling of four different HPMA-based polymers, the polymers were functionalized with dithiobenzoic ester end groups which can be reduced to free thiol moieties by tris(2-carboxyethyl)phosphine (TCEP) and covalently bind to arsenic (Figure 7).

Four different HPMA-based polymers were modified, three of them with a thiol content of 0.5%–1.0%. Another HPMA-polymer was especially designed to exhibit a higher thiol content of 10% by introducing additional disulfide sidechains (Figure 7). For radiolabeling, the radioarsenic was separated from the germanium target by distillation and after further work-up, ^72/74^As was available in PBS solution (pH 7) [62]. The different polymers were radiolabeled in aqueous solutions at 70 °C (low thiol) or 30 °C (high-thiol) and gave RCYs of ~20% for low-thiol-content polymers and up to 90% for the high-thiol-content polymer. The ^72/74^As-labeled HPMA-polymers were tested *in vitro* towards their stability. All new ^72/74^As-labeled HPMA-polymers showed an excellent stability in physiological saline over a time period of 48 h. The radiolabeled polymers were not further investigated or evaluated.

In similarity to McDevitt *et al.* [64], single walled nanotubes (SWNTs) were employed in combination with the longer-lived ^89^Zr by Ruggiero *et al.* [66]. The SWNTs were amino-functionalized and further derivatized with the chelator DFO for ^89^Zr-radiolabeling. Moreover, the SWNTs were coupled to an antiVE-cad (vascular endothelial cadherin) antibody (E4G10) for active targeting of neo-vascularization (angiogenesis). ^89^Zr-Radiolabeling was facilitated using ^89^Zr-oxalate at pH 5 for 60 min at 60 °C. The ^89^Zr-SWNTs were isolated via SEC. The new ^89^Zr-nanotubes were applied to *in vivo* PET studies using a human colon adenocarcinoma model (LS174T) in mice. PET imaging was performed daily over one week. The imaging studies revealed a fast blood clearance within one hour and a specific and high uptake in the targeted sites.

Choi *et al.* applied ^124^I to iron oxide NPs [67]. The magnetic radiolabeled NPs are intended to act as a dual-modality imaging agent for MRI/PET imaging of SLNs. Synthesized magnetic iron oxide NPs were highly monodispersed (σ < 5%) with a size of 15 nm. To facilitate the radioiodination, the surface of the NP was coated with serum albumin, of which the tyrosine groups offer the possibility of direct electrophilic iodination in the *ortho*-position. The hydrodynamic size of the albumin-coated particles was 32 nm. The radiolabeling was performed using [^124^I]NaI in presence of Iodo-Beads as oxidant. The ^124^I-labeled NPs were evaluated in Sprague-Dawley rats applying PET and MRI imaging with the focus on SLN detection. Using both modalities for functional and morphological information, a clear visualization of two brachial lymph nodes in rats was achieved. Furthermore, a dissection of the corresponding lymph node ducts in which the radiolabeled NPs were detected, confirmed the imaging results.

## 5. Conclusions

Radiolabeling approaches for NPs and polymers are essential tools in the development of potential drug delivery systems. In this regard, positron emitters are of special interest as they allow PET imaging. Furthermore, a radiolabeled analog of a drug delivery system for PET enables patient selection, therapy planning and monitoring by molecular imaging.

Several radiolabeling strategies with quite a variety of different PET isotopes have been developed and show very promising results. In principle, the very wide range of half-lives (see “*Clock-Of-Nuclides*”, Figure 2) allows tracking and detection of nanodimensional probes from the time point of administration to several days and even weeks p.i. Interestingly, several examples with short-lived isotopes (*i.e.*, ^18^F, ^68^Ga and even ^13^N) demonstrate the fundamental importance of the initial few hours. Moreover, short-lived isotopes have been used in pre-targeting concepts, which offer a great flexibility regarding the time point of imaging.

However, there are quite a number of problems one has to deal with during the evaluation of nanoparticular and polymeric systems for PET imaging. It starts with the synthesis of the structures and the decision of what labeling approach should be investigated or is suitable for the biological question asked (half-life; biological target). Subsequently, the radiosynthesis comes to the fore, meaning how to get the NPs or polymers labeled (direct labeling, prosthetic group labeling or *in situ* generation of the radionuclide) in sufficient RCYs and in the right formulation, like volume activity and/or purification. If all the physicochemical properties can be fulfilled, the *in vitro* and *in vivo* testing begins. *In vitro* tests of some QDs showed an increased toxicity, which could be decreased by using the known concept of PEGylation. It has also been shown that coating NPs with biocompatible/biodegradable structures has a positive effect on the *in vitro*/*in vivo* behavior. If the toxicity of the NPs and polymers could be excluded micro-PET studies were conducted.

The next step is the use of the radiolabeling and PET imaging platform for systematic optimization of NPs and polymers towards specific applications. A major aspect, which repeats throughout all *in vivo* evaluations of NPs or polymeric structures, is their accumulation in either the liver/spleen (large sizes) or the kidneys (small sizes). Noticeable is that not only the size of the particles matters, rather its architecture, which significantly determines the biodistribution. However, as long as new nanomaterials for potential drug delivery systems are developed, there is still a demand for new methods and strategies in radiolabeling of NPs and polymers to provide a suitable (dual)system for the asked biological question. Further research in this exciting field can be expected, ideally with a NP or polymer which accumulates specifically at target sites and can be tracked *in vivo*, and/or can transport a therapeutic agent.
